# A novel hypoxic tumor microenvironment signature for predicting the survival, progression, immune responsiveness and chemoresistance of glioblastoma: a multi-omic study

**DOI:** 10.18632/aging.103626

**Published:** 2020-08-28

**Authors:** Zihao Wang, Lu Gao, Xiaopeng Guo, Yaning Wang, Yu Wang, Wenbin Ma, Yi Guo, Bing Xing

**Affiliations:** 1 Department of Neurosurgery, Peking Union Medical College Hospital, Chinese Academy of Medical Sciences and Peking Union Medical College, Beijing 100730, P.R. China

**Keywords:** glioblastoma, prognostic model, hypoxic tumor microenvironment, immunotherapy, chemoresistance

## Abstract

The hypoxic tumor microenvironment (TME) was reported to promote the aggressive phenotype, progression, recurrence, and chemoresistance of glioblastoma (GBM). We developed and validated a hypoxia gene signature for individualized prognostic prediction in GBM patients. In total, 259 GBM-specific hypoxia-related genes (HRGs) were obtained in hypoxic cultured GBM cells compared with normoxic cells. By applying the k-means algorithm, TCGA GBM patients were divided into two subgroups, and the patients in Cluster 1 exhibited high HRG expression patterns, older age, and poor prognosis, which was validated in the CGGA cohort. Cox regression analyses were performed to generate an HRG-based risk score model consisting of five HRGs, which could reliably discriminate the overall survival (OS) and progression-free survival (PFS) of high- and low-risk patients in both the TCGA training and CGGA validation cohorts. Then, nomograms with the hypoxia signature for OS and PFS prediction were constructed for individualized survival prediction, better treatment decision-making, and follow-up scheduling. Finally, functional enrichment, immune infiltration, immunotherapy response prediction and chemotherapy resistance analyses demonstrated the vital roles of the hypoxic TME in the development, progression, multitherpy resistance of GBM. The hypoxia gene signature could serve as a promising prognostic predictor and potential therapeutic target to combat chemoresistant GBM.

## INTRODUCTION

Glioblastoma multiforme (GBM), corresponding to World Health Organization (WHO) grade IV glioma, is the most lethal and aggressive type of brain tumor, with a 5-year overall survival (OS) rate of approximately 5% and a median lifespan from diagnosis to death of approximately 15 months [1, 2]. Despite the remarkable progress made in the development of therapies for GBM, it still exhibits significant morbidity and mortality. With the rapid popularization of large-scale genome-sequencing technologies, numerous molecular biomarkers have been investigated for prognosis prediction, subgroup classification, risk stratification, and therapeutic targeting for cancers [3, 4]. However, due to the heterogeneous and invariable features of GBM, which are characterized by multiple genetic and epigenetic variations, separate biomarkers can present only limited value in predicting the prognosis of GBM patients in clinical application [3, 4]. Hence, explorations of the underlying molecular mechanisms and investigations of clinically applicable predictors for prognosis and therapeutic responses are indispensable for GBM patients.

Hypoxia is a common characteristic of solid tumors that is mainly due to the exuberant metabolic requirements of cancer cells exceeding the limit of the oxygen availability of the tumor [5]. The hypoxic tumor microenvironment (TME) has been reported to play a pivotal role in promoting a more aggressive phenotype and behavior of tumor cells, which thereby contributes to the progression, recurrence, chemoresistance and radioresistance of cancers [6, 7]. Emerging evidence has demonstrated that the hypoxic TME is associated with the poor prognosis of multiple cancers, especially GBM [6, 8]. A few studies have found that certain hypoxia-related genes (HRGs) and their mediators, hypoxia-inducible factors (HIFs), may serve as prognostic predictors and therapeutic targets in some cancers, such as colorectal cancer, breast cancer, and GBM [9, 10]. However, most studies mainly focused on single gene expression patterns regardless of the clinical setting, whereas a systematic analysis of the global gene expression patterns and comprehensive prognostic prediction models based on multiple HRGs have not been realized before in GBM [3, 4].

In this study, by performing a comprehensive multi-omic analysis based on transcriptomic, DNA methylation and copy number alteration (CNA) patterns, we aimed to develop and validate a hypoxic TME gene-based signature that could be applied for subgroup classification, risk stratification, prognosis prediction, and therapeutic targets for GBM patients. Then, novel promising nomograms for OS and progression-free survival (PFS) with favorable predictive performances were constructed and validated based on the hypoxia signature and clinicopathological features. Finally, gene set enrichment analysis (GSEA), immune infiltration analysis, immunotherapy response prediction, and chemotherapy resistance analysis of the HRGs were performed to investigate the vital roles of the hypoxic TME in the development, progression, immune responsiveness and chemoresistance of GBM.

## RESULTS

### Identification of GBM-Specific HRGs and enrichment analyses

**Figure 1:**
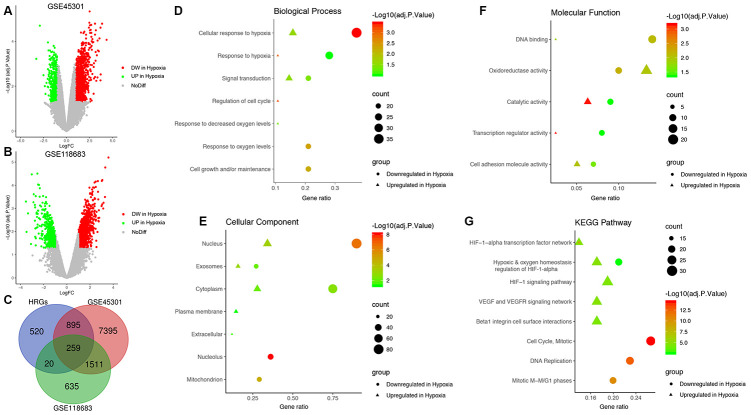
**Identification of glioblastoma (GBM)-specific hypoxia-related genes (HRGs) and enrichment analysis.** (**A**) Volcano plot of differentially expressed genes (DEGs) between normoxic and hypoxic cultured GBM cells in GSE45301. (**B**) Volcano plot of DEGs between normoxic and hypoxic cultured GBM cells in GSE118683. The vertical axis indicates the -log [adjusted P value (adj. P value)], and the horizontal axis indicates the log_2_ [fold change (FC)]. The red dots represent downregulated genes under hypoxic conditions, and the green dots represent upregulated genes under hypoxic conditions (adj. P value <0.01 and |log_2_(FC)|>1). (**C**) Venn diagram of the 259 GBM-specific HRGs, which are the genes in the intersection of the HRGs from MSigDB and the DEGs of GSE45301 and GSE118683. Biological processes (**D**), cellular components (**E**), molecular functions (**F**) and Kyoto Encyclopedia of Genes and Genomes (KEGG) pathways (**G**) enriched in the GBM-specific HRGs.

First, a total of 10,060 DEGs in GSE45301 and 2,425 DEGs in GSE118683 were identified between normoxic and hypoxic cultured GBM cells and were displayed in volcano plots (Figure 1A and 1B). Then, the 259 shared genes among the HRGs from MSigDB and the DEGs from GSE45301 and GSE118683 were determined as the GBM-specific HRGs, which were selected for further analysis (Figure 1C, Supplementary Table 2).

Enrichment analyses were performed on the GBM-specific HRGs to explore their corresponding molecular mechanisms in the tumorigenesis and progression of GBM. In the BP category, the HRGs were significantly enriched in response to hypoxia, and signal transduction (Figure 1D). In the CC category, the HRGs were significantly enriched in the nucleus and cytoplasm (Figure 1E). In the MF category, the HRGs were significantly enriched in DNA binding, oxidoreductase activity, and catalytic activity (Figure 1F). Moreover, KEGG pathway analysis demonstrated that the HRGs downregulated in hypoxic conditions were mainly enriched in hypoxic and oxygen homeostasis regulation of HIF-1-α, the cell cycle, and DNA replication, whereas the HRGs upregulated in hypoxic conditions were enriched in the HIF-1 signaling pathway, hypoxic and oxygen homeostasis regulation of HIF-1-α, VEGF and VEGFR signaling network, and beta-1 integrin cell surface interactions (Figure 1G).

### HRG-based molecular classification of GBM patients and associations with prognosis and clinicopathological characteristics

**Figure 2:**
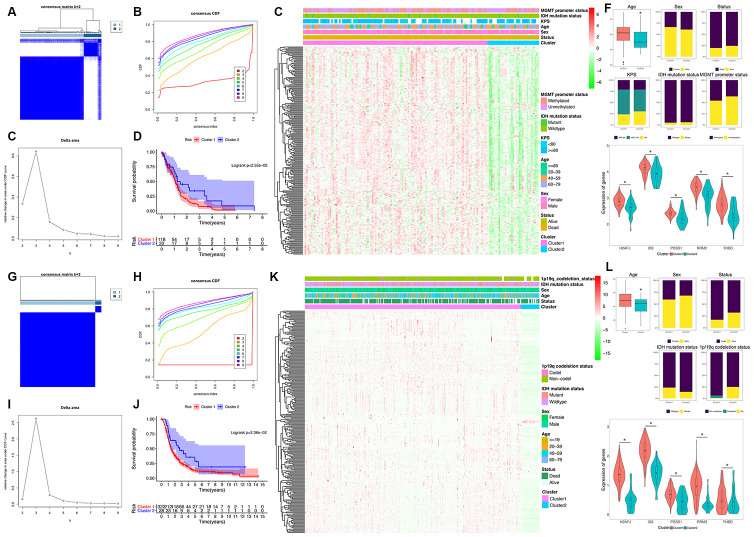
**Identification and validation of an HRG-based molecular classification of GBM patients using the unsupervised consensus clustering algorithm.** Consensus clustering matrix for k = 2, which was the optimal cluster number in the TCGA training cohort (**A**) and CGGA validation cohort (**G**). Cumulative distribution function (CDF) curves of the consensus score (k = 2-9) in the TCGA (**B**) and CGGA cohorts (**H**). The relative change in the area under the CDF curve (k = 2-9) in the TCGA (**C**) and CGGA cohorts (**I**). Kaplan-Meier (**K**–**M**) survival analyses of the patients in the Cluster 1 and Cluster 2 subgroups in the TCGA (**D**) and CGGA cohorts (**J**), which indicated that the patients in Cluster 1 had poorer OS than those in Cluster 2. The heatmap and clinicopathological features of the two clusters based on the expression patterns of the HRGs in the TCGA (**E**) and CGGA cohorts (**K**). The distributions of the clinicopathological factors and the expression patterns of the five HRGs included in the hypoxia signature between the two clusters of GBM patients in the TCGA (**F**) and CGGA cohorts (**L**). Upper and middle panel (**F** and **L**): Patients in Cluster 1 were older in both the training (P=0.024) and validation cohorts (P=0.047). No significant differences in the other clinicopathological factors were observed between the two clusters (all P>0.05). Bottom panel (**F** and **L**): The expression levels of the five HRGs were significantly higher in Cluster 1 than in Cluster 2 (all P<0.05) in both the training and validation cohorts. Asterisk means P<0.05 between two groups.

To explore a novel molecular classification of GBM based on the expression patterns of the HRGs, unsupervised consensus clustering was performed on the 151 TCGA GBM patients. According to the relative change in the area under the CDF curve and consensus heatmap, the optimal number of clusters was determined as two (k value = 2), and no appreciable increase was observed in the area under the CDF curve (Figure 2A–2C). Then, all 151 patients were divided into two subgroups, including 118 (78.1%) patients in Cluster 1, and 33 (21.9%) in Cluster 2. K-M survival analysis demonstrated that patients in Cluster 1 showed significantly worse OS than those in Cluster 2 (log-rank P=2.55×10^-2^; Figure 2D). Then, the same method was applied to validate the molecular classification in the CGGA GBM patients. As shown in Figure 2G–2I, the optimal number of clusters was also determined as two (k value = 2), and the 350 GBM patients were divided into Cluster 1 (322 patients, 92.0%) and Cluster 2 (28 patients, 8.0%). The patients in Cluster 1 also showed significantly worse OS than those in Cluster 2 (log-rank P=2.36×10^-2^; Figure 2J). Then, the cluster quality measure was applied to verify the similarities between the different subgroups. The IGP score of TCGA Cluster 1 was 0.752 and that of TCGA Cluster 2 was 0.235 (P<0.001), whereas the IGP score of CGGA Cluster 1 was 0.791 and that of CGGA Cluster 2 was 0.250 (P<0.001). There was no significant difference between TCGA Cluster 1 and CGGA Cluster 1 (P=0.215) nor between TCGA Cluster 2 and CGGA Cluster 2 (P=0.611).

Then, we also analyzed the expression patterns of the HRGs and distributions of the clinicopathological factors between two clusters of GBM patients. The expression patterns of the GBM-specific HRGs were visualized in the heatmaps shown in Figure 2E (TCGA) and Figure 2K (CGGA). Generally, the expression levels of most HRGs in Cluster 1 were significantly upregulated compared with those in Cluster 2 in both the TCGA and CGGA GBM cohorts, which indicated that an increase in the expression levels of the HRGs was associated with poor prognosis. The expression levels of the five genes that were included the hypoxia signature were significantly more highly expressed in Cluster 1 than in Cluster 2 (all P<0.05) in both the TCGA and CGGA GBM cohorts (Figure 2F and 2L). Compared with that in Cluster 2, patients in Cluster 1 were older in both the training (P=0.024) and validation cohorts (P=0.047). However, no significant difference in the other clinicopathological factors was observed between the two clusters (all P>0.05, Figure 2F and 2L). Overall, the patients in the Cluster 1 subgroup, with high expression patterns of HRGs and older age, commonly exhibited poor prognosis. These findings demonstrated that our novel HRG-based molecular classification of GBM was robust and reliable in different populations, and different survival outcomes and clinicopathological parameters can be clearly discriminated.

### Generation and validation of the hypoxia signature

Univariate Cox regression analysis was performed on the 259 GBM-specific genes in the TCGA training cohort and identified 19 prognosis-associated HRGs. Then, LASSO regression (Supplementary Figure 1A, 1B) followed by multivariate Cox regression (Supplementary Figure 1C) analysis were performed to further screen the genes with the most significant prognostic value. Finally, five HRGs, including thrombomodulin (THBD, HR=2.45), inhibitor of DNA binding 3 (ID3, HR=0.27), decaprenyl diphosphate synthase subunit 1 (PDSS1, HR=0.17), H2A histone family member J (H2AFJ, HR=3.35), and ribonucleotide reductase regulatory subunit M2 (RRM2, HR=3.10), were selected as the significant prognostic genes (Supplementary Figure 1C).

**Figure 3:**
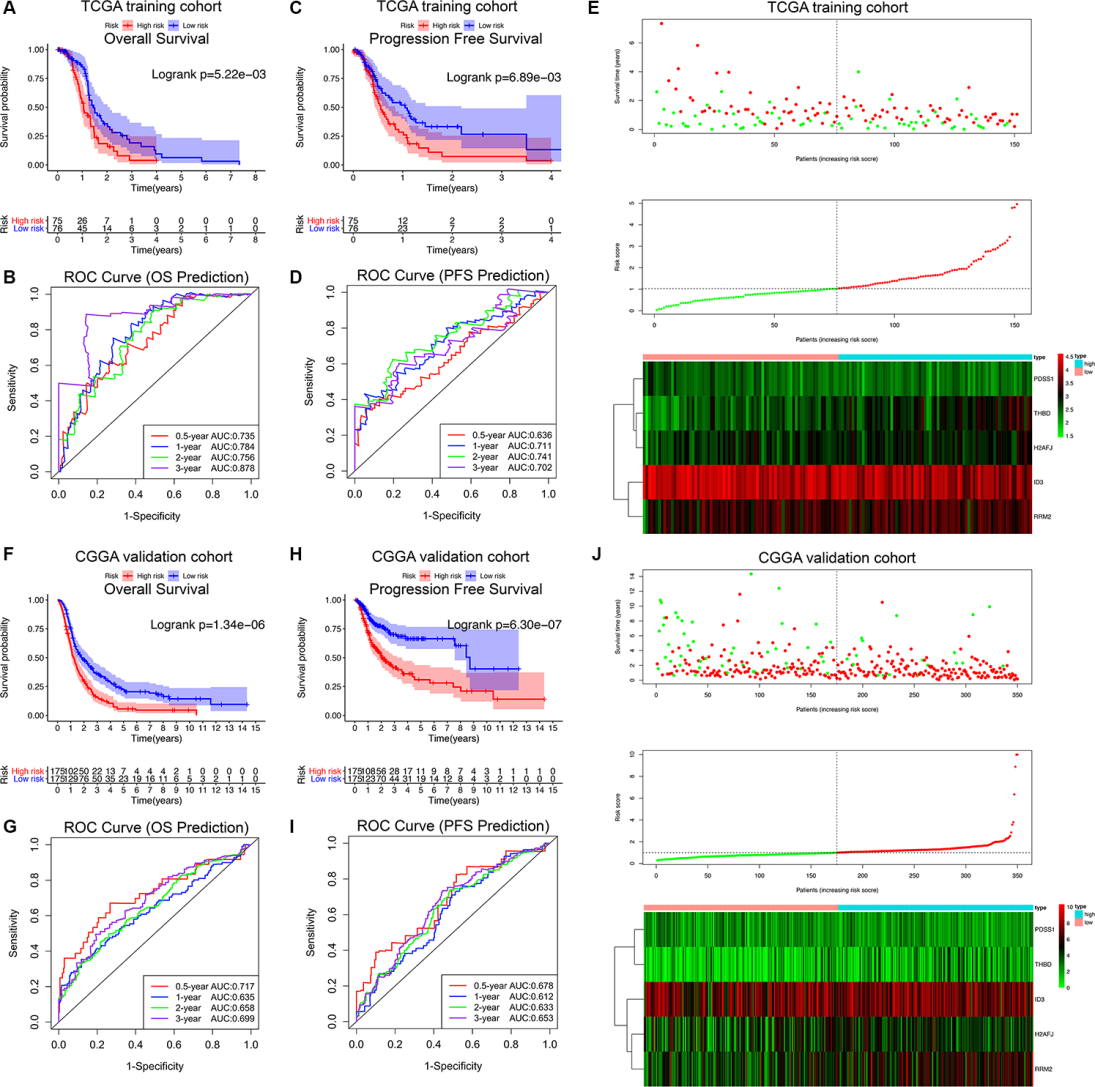
**Survival analysis, prognostic performance and risk score analysis of the HRG-based risk score model in GBM patients.** K-M survival analysis was performed to estimate the overall survival (OS) of high-risk and low-risk patients in the TCGA training cohort (**A**) and CGGA validation cohort (**F**). Additionally, K-M survival analysis was also performed to estimate the progression-free survival (PFS) of high-risk and low-risk patients in the TCGA (**C**) and CGGA cohorts (**H**). The high-risk groups had significantly poorer OS and PFS rates than the low-risk groups. The prognostic performance of the hypoxia signature demonstrated by the time-dependent ROC curve for predicting the 0.5-, 1-, 2-, and 3-year OS rates in the TCGA (**B**) and CGGA cohorts (**G**). The prognostic performance of the hypoxia signature demonstrated by the time-dependent ROC curve for predicting the 0.5-, 1-, 2-, and 3-year PFS rates in the TCGA (**D**) and CGGA cohorts (**I**). Risk score analysis of the hypoxia signature in the TCGA (**E**) and CGGA cohorts (**J**). Upper panel (**I** and **J**): Patient survival status and time distributed by risk score. Middle panel (**I** and **J**): Risk score curves of the hypoxia signature. Bottom panel (**I** and **J**): Heatmaps of the expression levels of the 5 HRGs in the GBM samples. The colors from green to red indicate the expression level from low to high.

The HRG-based prognostic risk score model was established with the following formula: Risk score = Exp_THBD_ × 0.894 + Exp_ID3_ × (-1.298) + Exp_PDSS1_ × (-1.782) + Exp_H2AFJ_ × 1.208 + Exp_RRM2_ × 1.133. The risk score was calculated for each patient in the TCGA training cohort, and all patients were divided into a high-risk (high risk score) and low-risk (low risk score) group using the median value of the risk score as the cutoff (Figure 3E). Survival analysis demonstrated that compared with low-risk patients, high-risk patients showed markedly poorer OS (log-rank P = 5.22×10^-3^; Figure 3A) and PFS (log-rank P = 6.89×10^-3^; Figure 3C). The C-index of the hypoxia signature was 0.801 (95% CI, 0.762 to 0.840; P = 6.91×10^-21^) for OS prediction and 0.759 (95% CI, 0.720 to 0.798; P = 2.21×10^-15^) for PFS prediction. In addition, by performing time-dependent ROC analysis, the hypoxia signature showed excellent values in predicting 0.5-, 1-, 2- and 3-year OS rates, with respective AUC values of 0.735, 0.784, 0.756 and 0.878 in the TCGA GBM training set (Figure 3B). Moreover, the AUCs for the 0.5-, 1-, 2-, and 3-year PFS rates with the prognostic model were 0.636, 0.711, 0.741, and 0.702, respectively (Figure 3D).

Finally, the predictive ability of the hypoxia signature was further validated in the different patient populations from CGGA dataset in a similar way. As shown in Figure 3J, all 350 GBM patients were classified into high-risk and low-risk groups utilizing the risk score formula mentioned earlier based on the median value as the cutoff. Consistent with the above findings, K-M survival analysis demonstrated that patients with high risk scores in the validation set also had a significantly shorter OS (log-rank P = 1.34×10^-6^; Figure 3F) and PFS (log-rank P = 6.30×10^-7^; Figure 3H) than those with low risk scores. The time-dependent ROC analysis also suggested favorable values in predicting both OS and PFS in the CGGA validation set (Figure 3G–3I). These results indicated that the hypoxia signature may serve as a robust and reliable prognostic predictor for both the OS and PFS of GBM patients from different populations.

### Construction and validation of the prognostic nomogram for OS prediction

Table 1 shows the demographics and clinicopathological features of the GBM patients in the TCGA training cohort and CGGA validation cohort based on the hypoxia signature. To investigate whether the prognostic significance of the hypoxia signature is independent of other clinicopathological parameters in predicting the OS of GBM patients, univariate and multivariate Cox regression analyses were performed, which demonstrated that the hypoxia signature (HR 1.435, P = 1.55×10^-3^) was significantly associated with OS in the TCGA training set (Table 2). Moreover, in the CGGA validation cohort, the hypoxia signature (HR 1.098, P = 1.78×10^-10^) was also proven to be a significant independent prognostic predictor for OS (Table 2).

**Table 1:** Demographics and clinicopathological characteristics of GBM patients in the TCGA training cohort and CGGA validation cohort based on the hypoxia signature.

**Variables**	**TCGA cohort (Training set)**			**CGGA cohort (Validation set)**		
	**Total (n=151)**	**Low risk (n=76)**	**High risk (n=75)**	**Total (n=350)**	**Low risk (n=175)**	**High risk (n=175)**
**Age (years)**	59.6±13.7	58.8±13.4	60.4±14.0	48.1±13.3	47.2±13.1	48.9±13.5
**Sex**						
**Female**	53	21	32	139	63	76
**Male**	98	55	43	211	112	99
**KPS**						
**< 80**	32	15	17	NA		
**>= 80**	81	41	40	NA		
**NA**	38	20	18	NA		
**Pharmacotherapy**					
**TMZ**	64	37	27	61 (No)	24	37
**TMZ+BEV**	26	10	16	269 (Yes)	139	130
**Others (No TMZ)**	19	10	9	-	-	-
**No or NA**	42	19	23	20 (NA)	12	8
**Radiotherapy**						
**No**	22	11	11	48	19	29
**Yes**	122	63	59	283	146	137
**NA**	7	2	5	19	10	9
**Surgery**						
**Biopsy only**	16	10	6	NA		
**Tumor resection**	135	66	69	NA		
**IDH status**						
**Wildtype**	147	68	75	270	113	157
**Mutant**	8	8	0	80	62	18
**MGMT promoter status**					
**Methylated**	66	30	36	NA		
**Unmethylated**	85	46	39	NA		
**TERT status**						
**Wildtype**	146	73	73	NA		
**Mutant**	5	3	2	NA		
**BRAF status**						
**Wildtype**	146	74	72	NA		
**Mutant**	5	2	3	NA		
**ATRX status**						
**Wildtype**	140	68	72	NA		
**Mutant**	11	8	3	NA		
**EGFR status**						
**Wildtype**	97	42	55	NA		
**Mutant**	54	34	20	NA		
**1p/19q status**						
**Non-codeletion**	NA			323	152	171
**Codeletion**	NA			17	15	2
**NA**	NA			10	8	2

GBM, glioblastoma; NA, not available; KPS, Karnofsky performance score; TMZ, temozolomide; BEV, bevacizumab; PCV, procarbazine lomustine vinCRISTine.

“Others (No TMZ)” in pharmacotherapy included PCV, PCV+BEV, and other drugs, including avastin, carmustine, and irinotecan.

**Table 2:** Univariate and multivariate cox proportional hazards analysis of clinicopathological variables and hypoxia signature based on overall survival (OS) in the TCGA GBM training cohort and CGGA GBM validation cohort.

**OS Prediction Model Variables**	**TCGA training cohort (N=151)**				**CGGA validation cohort (N=350)**			
	**Univariate Analysis**		**Multivariate analysis**		**Univariate Analysis**		**Multivariate analysis**	
	**HR (95% CI)**	**P value**	**HR (95% CI)**	**P value**	**HR (95% CI)**	**P value**	**HR (95% CI)**	**P value**
Age	1.028(1.013-1.044)	**1.98e-04**	1.021(1.003-1.039)	**2.23e-02**	1.078(1.048-1.108)	**8.35e-05**	1.061(1.030-1.091)	**1.97e-02**
Sex (Female/Male)	0.916(0.626-1.341)	0.65	-	-	1.063(0.837-1.350)	0.61	-	-
KPS (<80/>=80/NA)	0.926(0.696-1.233)	0.59	-	-	NA		NA	
Pharmacotherapy (TMZ/ TMZ+BEV/Others (No TMZ)/No or NA)	0.883(0.852-0.913)	**1.06e-04**	0.884(0.779-0.989)	**1.31e-02**	0.573(0.432-0.759)	**1.04e-04**	0.600(0.441-0.817)	**1.17e-03**
Radiotherapy (No/Yes/NA)	0.433(0.262-0.714)	**1.04e-03**	0.314(0.183-0.538)	**2.44e-05**	0.668(0.492-0.908)	**9.96e-03**	0.782(0.752-0.812)	**1.64e-02**
Surgery (Biopsy only/ Tumor resection)	0.934(0.523-1.667)	0.82	-	-	NA		NA	
IDH status (Wildtype/Mutant)	0.262(0.096-0.715)	**8.91e-03**	0.494(0.389-0.599)	**4.05e-02**	0.752(0.566-0.988)	**3.89e-02**	0.772(0.742-0.802)	**4.76e-02**
MGMT promoter status (Methylated/Unmethylated)	1.434(1.133-1.733)	**6.84e-03**	1.359(1.254-1.464)	**1.42e-02**	NA		NA	
TERT promoter status (Wildtype/Mutant)	0.906(0.287-2.861)	0.87	-	-	NA		NA	
BRAF status (Wildtype/Mutant)	1.973(0.720-5.410)	0.19	-	-	NA		NA	
ATRX status (Wildtype/Mutant)	0.426(0.187-0.973)	**4.28e-02**	0.917(0.235-3.580)	**0.91**	NA		NA	
EGFR status (Wildtype/Mutant)	1.273(0.873-1.857)	0.21	-	-	NA		NA	
1p/19q status (Non-codeletion/Codeletion/NA)	NA		NA		0.913 (0.662-1.259)	0.58	-	-
Hypoxia signature	1.507(1.252-1.815)	**1.49e-05**	1.435(1.147-1.795)	**1.55e-03**	1.092(1.062-1.123)	**7.60e-10**	1.098(1.067-1.130)	**1.78e-10**

OS, overall survival; GBM, glioblastoma; NA, not available; HR, hazard ratio; CI, confidence interval; KPS, Karnofsky performance score; TMZ, temozolomide; BEV, bevacizumab; PCV, procarbazine lomustine vinCRISTine.

“Others (No TMZ)” in pharmacotherapy included PCV, PCV+BEV, and other drugs, including avastin, carmustine, and irinotecan.

All statistical tests were two-sided. Bold type means P<0.05.

**Figure 4:**
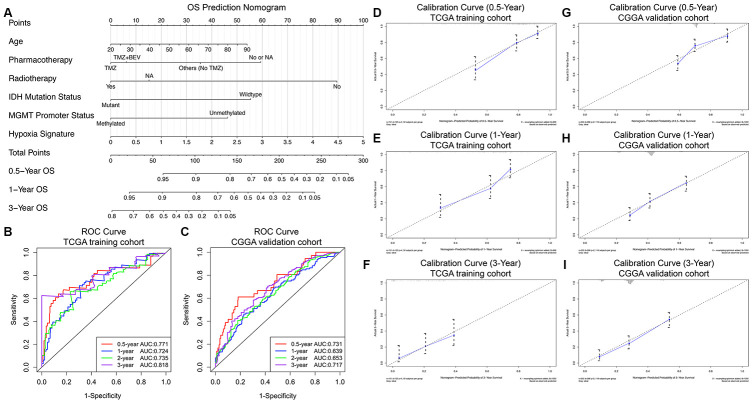
**Prognostic nomogram to predict the 0.5-, 1-, and 3-year OS probabilities of GBM patients.** (**A**) Nomogram model to predict the survival of GBM patients based on the TCGA training cohort. The prognostic performance of the prognostic nomogram demonstrated by the ROC curve for predicting the 0.5-, 1-, and 3-year OS rates in the TCGA training cohort (**B**) and CGGA validation cohort (**C**). Calibration curves of the prognostic nomogram for predicting OS at 0.5, 1, and 3 years in the TCGA (**D**–**F**) and CGGA (**G**–**I**) cohorts. The actual survival is plotted on the y-axis, and the nomogram-predicted probability is plotted on the x-axis.

Finally, a prognostic nomogram for GBM patients was successfully constructed in order to provide a clinically applicable quantitative approach for individual OS prediction. Age, pharmacotherapy, radiotherapy, IDH mutation status, MGMT promoter methylation status, and the hypoxia signature were integrated into the final OS prediction model (Figure 4A). The C-index of the prognostic nomogram was 0.822 (95% CI, 0.783 to 0.861; P = 2.99×10^-20^). The time-dependent ROC analysis indicated favorable predictive abilities of the 0.5-, 1-, 2- and 3-year OS rates, with AUC values of 0.771, 0.724, 0.735 and 0.818, respectively (Figure 4B). The calibration plots showed excellent agreement between the predicted 0.5-, 1- and 3-year OS rates and the actual observations in the TCGA cohort (Figure 4D–4F). Additionally, in the CGGA validation cohort, the C-index of the nomogram for predicting the OS of the 350 GBM patients was 0.751 (95% CI, 0.712 to 0.790; P = 1.79×10^-13^). The time-dependent AUCs for the 0.5-, 1-, 2-, and 3-year OS rates with the prognostic nomogram were 0.731, 0.639, 0.653, and 0.717, respectively, in the CGGA validation cohort (Figure 4C). The calibration plots also showed excellent agreement between the OS predictions and the actual observations for the probabilities of 0.5-, 1- and 3-year survival in the validation set (Figure 4G–4I). All these findings suggested the appreciable reliability of the prognostic nomogram for OS prediction.

### Construction and validation of the progression nomogram for PFS prediction

**Figure 5:**
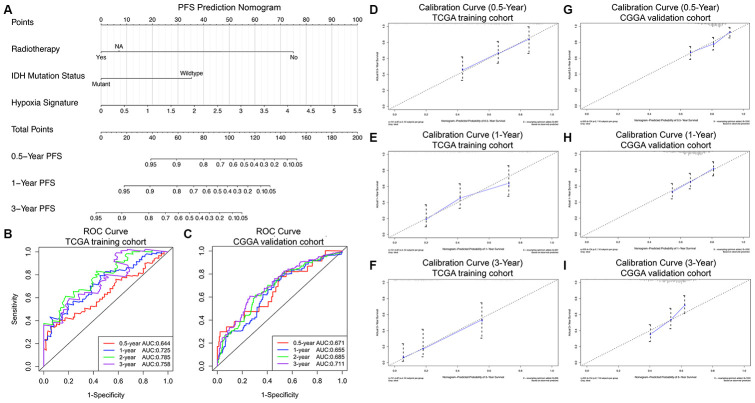
**Progression nomogram to predict the 0.5-, 1-, and 3-year PFS probabilities of GBM patients.** (**A**) Nomogram model to predict the survival of GBM patients based on the TCGA training cohort. The prognostic performance of the progression nomogram demonstrated by the ROC curve for predicting the 0.5-, 1-, and 3-year PFS rates in the TCGA training cohort (**B**) and CGGA validation cohort (**C**). Calibration curves of the prognostic nomogram for predicting PFS at 0.5, 1, and 3 years in the TCGA (**D**–**F**) and CGGA (**G**–**I**) cohorts. The actual survival is plotted on the y-axis, and the nomogram-predicted probability is plotted on the x-axis.

Consistent with the methods of constructing the prognostic nomogram, univariate and multivariate Cox regression analyses were sequentially performed, and the results suggested that the hypoxia signature was a significant independent prognostic predictor for PFS in both the TCGA training set and CGGA validation set (Table 3). Then, the progression nomogram for PFS prediction was constructed based on radiotherapy, IDH mutation status, and the hypoxia signature (Figure 5A). The C-index of the progression nomogram was 0.763 (95% CI, 0.724 to 0.802; P = 4.55×10^-31^). The time-dependent ROC analysis indicated favorable predictive abilities of the 0.5-, 1-, 2- and 3-year PFS rates, with AUC values of 0.644, 0.725, 0.785 and 0.758, respectively (Figure 5B). The calibration plots showed excellent agreement between the predicted 0.5-, 1- and 3-year PFS rates and actual observations in the TCGA cohort (Figure 5D–5F). In addition, in the CGGA validation cohort, the C-index of the progression nomogram for predicting the PFS of the 350 GBM patients was 0.715 (95% CI, 0.676 to 0.754; P = 5.13×10^-20^). The time-dependent AUCs for the 0.5-, 1-, 2-, and 3-year PFS rates with the progression nomogram were 0.671, 0.655, 0.685, and 0.711, respectively, in the CGGA validation cohort (Figure 5C). The calibration plots also showed excellent agreement between the PFS predictions and actual observations for the probabilities of 0.5-, 1- and 3-year survival in the validation cohort (Figure 5G–5I). All these findings demonstrated the appreciable reliability of the progression nomogram for PFS prediction.

**Table 3:** Univariate and multivariate cox proportional hazards analysis of clinicopathological variables and hypoxia signature based on progression free survival (PFS) in the TCGA GBM training cohort and CGGA GBM validation cohort.

**PFS Prediction Model Variables**	**TCGA training cohort (N=151)**				**CGGA validation cohort (N=350)**			
	**Univariate Analysis**		**Multivariate analysis**		**Univariate Analysis**		**Multivariate analysis**	
	**HR (95% CI)**	**P value**	**HR (95% CI)**	**P value**	**HR (95% CI)**	**P value**	**HR (95% CI)**	**P value**
Age	1.003(0.988-1.018)	0.69	-	-	1.179(0.967-1.991)	0.54	-	-
Sex (Female/Male)	1.282(0.808-2.034)	0.29	-	-	1.001(0.708-1.416)	0.99	-	-
KPS (<80/>=80/NA)	0.927(0.675-1.272)	0.64	-	-	NA		NA	
Pharmacotherapy (TMZ/ TMZ+BEV/Others (No TMZ)/No or NA)	0.821(0.776-1.093)	0.35	-	-	1.005(0.648-1.558)	0.98	-	-
Radiotherapy (No/Yes/NA)	0.802(0.772-0.832)	**4.60e-04**	0.822(0.783-0.854)	**2.82e-02**	0.841(0.802-0.881)	**4.55e-05**	0.881(0.868-0.893)	**3.13e-03**
Surgery (Biopsy only/ Tumor resection)	0.954(0.491-1.852)	0.89	-	-	NA		NA	
IDH status (Wildtype/Mutant)	0.188(0.046-0.774)	**2.06e-02**	0.458(0.108-0.850)	**2.91e-02**	0.664(0.464-0.949)	**2.46e-02**	0.828(0.569-0.867)	**3.24e-02**
MGMT promoter status (Methylated/Unmethylated)	1.320(0.853-2.044)	0.21	-	-	NA		NA	
TERT promoter status (Wildtype/Mutant)	1.037(0.252-4.266)	0.96	-	-	NA		NA	
BRAF status (Wildtype/Mutant)	1.641(0.398-6.756)	0.49	-	-	NA		NA	
ATRX status (Wildtype/Mutant)	0.488(0.197-1.210)	0.12	-	-	NA		NA	
EGFR status (Wildtype/Mutant)	1.419(0.909-2.216)	0.12	-	-	NA		NA	
1p/19q status (Non-codeletion/Codeletion/NA)	NA		NA		1.051 (0.684-1.615)	0.82	-	-
Hypoxia signature	2.103(1.648-2.683)	**2.32e-09**	1.993(1.542-2.576)	**1.38e-07**	1.048(1.031-1.066)	**1.36e-08**	1.045(1.028-1.063)	**1.57e-07**

PFS, progression free survival; GBM, glioblastoma; NA, not available; HR, hazard ratio; CI, confidence interval; KPS, Karnofsky performance score; TMZ, temozolomide; BEV, bevacizumab; PCV, procarbazine lomustine vinCRISTine.

“Others (No TMZ)” in pharmacotherapy included PCV, PCV+BEV, and other drugs, including avastin, carmustine, and irinotecan.

All statistical tests were two-sided. Bold type means P<0.05.

### Expression, survival and GSEA analyses of the five HRGs

**Figure 6:**
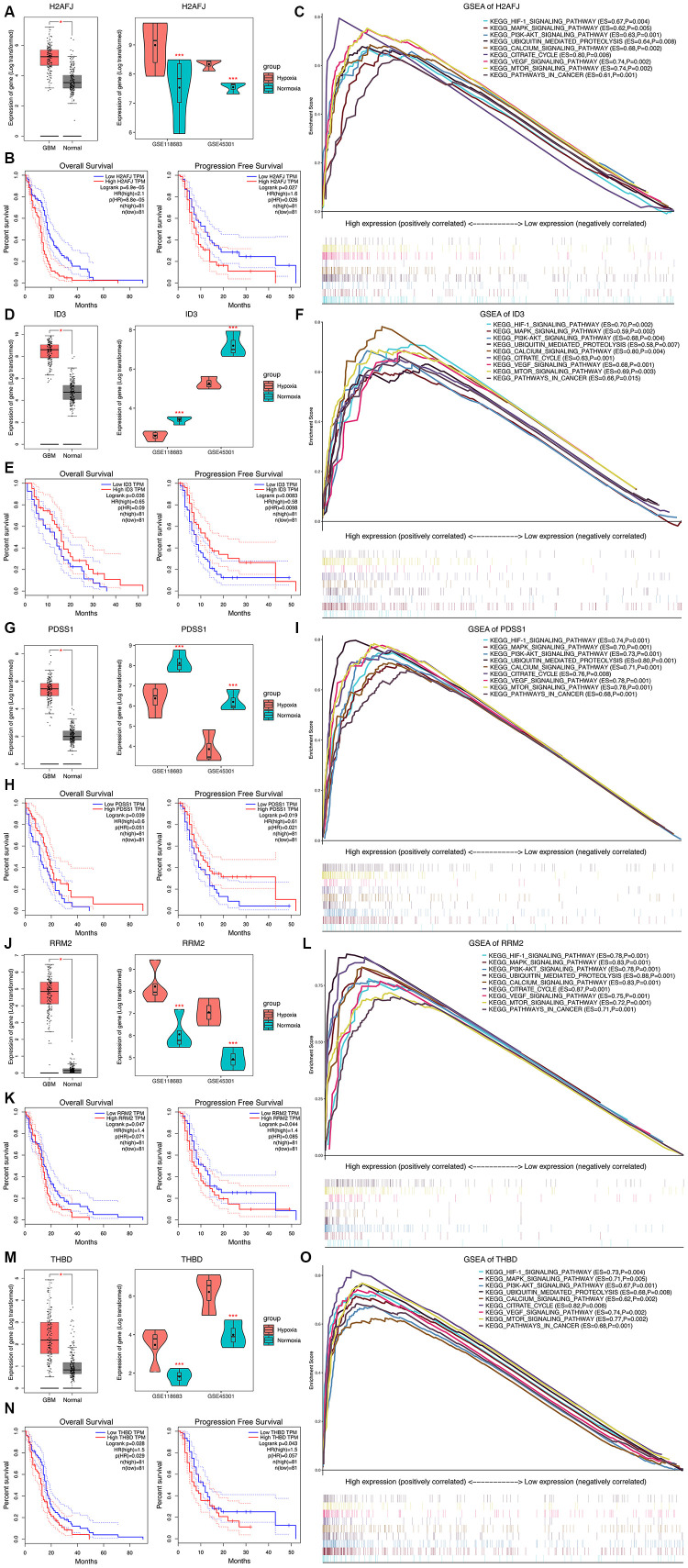
**Expression analysis, survival analysis, and gene set enrichment analysis (GSEA) of the 5 HRGs in the hypoxia signature.** Expression analysis of H2AFJ (**A**), ID3 (**D**), PDSS1 (**G**), RRM2 (**J**), and THBD (**M**). Left panel: Expression levels of the 5 HRGs in 163 GBM samples and 207 normal samples. Right panel: Expression levels of the 5 HRGs in hypoxia and normoxia cultured GBM cells from GSE118683 and GSE45301. Red asterisks mean P < 0.05 between two groups. (**K**–**M**) survival analysis of H2AFJ (**B**), ID3 (**E**), PDSS1 (**H**), RRM2 (**K**), and THBD (**N**). Left panel: K-M survival analysis was performed to estimate the OS of GBM patients with high and low expression levels of the corresponding HRG. Right panel: (**K**–**M**) survival analysis was performed to estimate the PFS of GBM patients with high and low expression levels of the corresponding HRG. GSEA of H2AFJ (**C**), ID3 (**F**), PDSS1 (**I**), RRM2 (**L**), and THBD (**O**) in the TCGA GBM cohort. The enriched KEGG pathways of the 5 HRGs are listed in the upper right. ES, enrichment score; P, nominal P value.

The expression levels of the 5 most significant prognostic HRGs between GBM and normal tissues were further validated in the Gene Expression Profiling Interactive Analysis (GEPIA) database, including 163 GBM and 207 normal samples [11]. We found that all the 5 HRGs were overexpressed in GBM tissues compared with normal tissues (Figure 6A, 6D, 6G, 6J, 6M, left panels). In addition, H2AFJ (Figure 6A, right panel), RRM2 (Figure 6J, right panel), and THBD (Figure 6M, right panel) had high expression in cells cultured under a hypoxic environment, whereas ID3 (Figure 6D, right panel) and PDSS1 (Figure 6G, right panel) had low expression in cells cultured under a hypoxic environment compared with that in cells cultured under a normoxia environment. Notably, K-M survival analyses demonstrated that high expression of H2AFJ (Figure 6B), RRM2 (Figure 6K), and THBD (Figure 6N) was associated with poor OS and PFS, while low expression of ID3 (Figure 6E) and PDSS1 (Figure 6H) was associated with poor OS and PFS. Hence, because solid tumors, such as GBM, grow under a hypoxic TME, we believe that hypoxia could serve as an important contributing factor for the poor prognosis of GBM patients by regulating the expression levels of the prognostic HRGs.

GSEA revealed that high expression levels of the 5 genes were significantly enriched in the KEGG pathways related to hypoxia and the development of tumors, including the HIF-1 signaling pathway, PI3K-AKT signaling pathway, MAPK signaling pathway, mTOR signaling pathway, pathways in cancer, apoptosis, and the cell cycle (Figure 6C, 6F, 6I, 6L, 6O). These findings strongly suggested the potential roles of the hypoxic TME in the tumorigenesis and progression of GBM, which may provide new evidence for cancer-targeted treatments involving the prognostic HRGs.

### Immune infiltration analysis of the five HRGs

**Figure 7:**
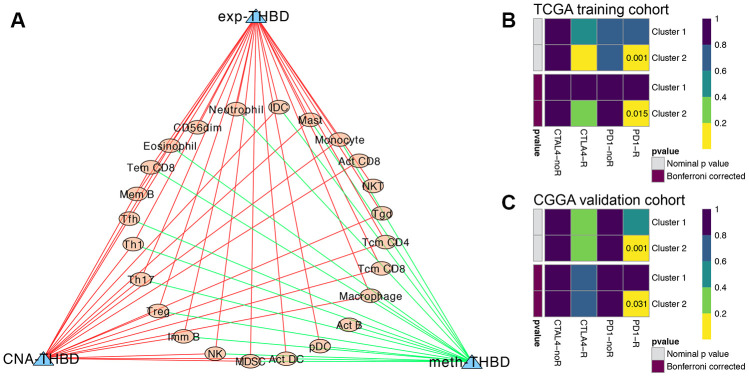
**Immune infiltration analysis and immunotherapy response predictions.** (**A**) The regulatory network between TILs and the expression, methylation and CNA of THBD, with |Pearson correlation coefficient| > 0.3, and P < 0.05. Yellow dots represent TILs of GBM, and blue triangles represent THBD. Green/Red lines represent negative/positive correlations between TILs and THBD. Subclass mapping analysis of the TCGA (**B**) and CGGA (**C**) GBM patients for predicting the likelihood of clinical response to anti-PD1 and anti-CTLA4 therapy in different clusters based on the novel HRG-based classification. R was short for immunotherapy respondent.

Then, correlation analyses between the TIL patterns of GBM and the HRGs were further investigated. As shown in Supplementary Figure 2, the expression levels of H2AFJ were positively correlated with innate immune cells and CD4^+^ and CD8^+^ T-cell subsets; the expression levels of RRM2 were positively correlated with Act CD4 cells and negatively correlated with Th cells, B cells, and DCs; and the expression levels of THBD were positively correlated with almost all the immune cells (78.6%) infiltrated in GBM tumors. Interestingly, both THBD and H2AFJ were significantly positively correlated with the main tumor-associated immunosuppressive cells, including Treg cells, MDSCs, NK cells and macrophages (Supplementary Figure 2). In terms of the methylation patterns of the HRGs, the methylation of ID3 was positively correlated with CD4^+^ T-cell subsets, MDSCs and DCs, and the methylation of THBD was negatively correlated with most of the immune cells (60.7%). For the CNAs of the HRGs, THBD was the only gene whose CNA levels were significantly positively correlated with 18 (64.3%) types of TILs. Hence, THBD was believed to be the most vital HRG in regulating the infiltration patterns of immune cells in GBM and thereby promoting the immunosuppressive TME of GBM (Figure 7A). Therefore, we believe that hypoxia might promote the immunosuppressive microenvironment of GBM mediated by HRGs.

### Predictions of immunotherapy response of the GBM patients

TIDE algorithm was applied to predict the likelihood of immunotherapy response of each HRG-based molecular clusters of GBM patients. In the TCGA training cohort, Cluster 2 (54.5%, 18/33) patients were more likely to respond to immunotherapy than Cluster 1 (24.6%, 29/118) (P = 0.001). Similarly, in the CGGA validation cohort, Cluster 2 (53.6%, 15/28) patients were also more sensitive to immunotherapy than Cluster 1 (26.7%, 86/322) (P = 0.003). Then, subclass mapping analysis was further used to predict the likelihood of clinical response to anti-PD1 and anti-CTLA4 therapy of the two clusters. Submap analysis demonstrated that compared with Cluster 1 GBM patients, Cluster 2 patients in both TCGA and CGGA cohort could be more sensitive to PD1 inhibitors, with Bonferroni-corrected P = 0.015 and 0.031, respectively (Figure 7B, and 7C).

### Chemotherapy resistance analysis of the five HRGs

As shown in Supplementary Figure 3, the expression levels of H2AFJ, RRM2, and THBD were positively correlated with drug resistance, whereas those of ID3 and PDSS1 were negatively correlated with drug resistance. For instance, high expression levels of H2AFJ, RRM2 and THBD and low expression levels of PDSS1 were highly resistant to temozolomide (TMZ), and high expression levels of H2AFJ and THBD and low expression levels of PDSS1 were highly resistant to BRD-A05715709 and BRD-A71883111, which are inhibitors of IDH1 R132H. Moreover, high expression levels of RRM2 and THBD were highly resistant to lomeguatrib, an inhibitor of MGMT. However, as mentioned earlier, hypoxia contributed to the increased expression levels of H2AFJ, RRM2, and THBD and decreased expression levels of ID3 and PDSS1, which thereby would lead to the enhancement of drug resistance to chemotherapy. Hence, we believe that the hypoxic TME might mediate and promote chemoresistance by regulating the expression levels of the corresponding HRGs. Targeted drugs that can regulate the expression levels of these HRGs might be combined with chemotherapy drugs such as TMZ or bevacizumab to possibly improve or even reverse chemoresistance in GBM patients. Our study not only indicated HRGs as predictors of prognosis and immunotherapy and chemotherapy effectiveness but also suggested a new treatment strategy to combat chemoresistant GBM by targeting those HRGs.

## DISCUSSION

GBM is the most malignant and aggressive intracranial solid tumor and is commonly characterized by severely low tumor oxygenation, referred to as a hypoxic TME. As reported by recent studies, the hypoxic TME has been shown to play a vital role in promoting the aggressive phenotypes and invasive behaviors of malignancies [6, 7]. Emerging evidence has demonstrated that hypoxia is associated with the failure of conventional cancer therapies and poor prognosis of multiple cancers, especially GBM [6, 8]. Under the critical regulations of the hypoxic TME, HRGs and HIFs are involved in different tumoral mechanisms of GBM, such as differentiation, angiogenesis, genomic instability, resistance to therapies, invasion and metastasis [6–8]. Huang et al. [12] reported that the HIF-1α/miR-224-3p/ATG5 axis can affect the cell mobility and chemosensitivity regulated by hypoxia in GBM and astrocytoma. Ahmed et al. [13] found that hypoxia contributed to the upregulated expression of CD133 and enhanced resistance to cisplatin, TMZ and etoposide in GBM in vitro models. Hence, HRGs can be widely used as promising prognostic predictors and therapeutic targets for GBM. However, there is still a lack of systematic analyses of the global gene expression patterns and comprehensive prognostic prediction models based on multiple HRGs for GBM.

In this study, we first developed a novel molecular classification of GBM patients based on the expression patterns of GBM-specific HRGs, which was then validated by the CGGA dataset. The patients in the Cluster 1 subgroup, with high expression patterns of the HRGs and older age, commonly exhibited poor prognosis. These results demonstrated that GBM patients from different populations can be reliably classified into two subgroups based on different hypoxic TME gene signatures. Then, Cox and LASSO regression analyses were sequentially performed to identify the prognosis-associated HRGs. Expression and survival analyses of the five HRGs demonstrated that hypoxia could contribute to the poor prognosis of GBM patients by regulating the expression levels of the prognostic HRGs, which was consistent with the findings of previous studies [12, 13]. GSEA further revealed that high expression levels of the 5 genes were significantly enriched in the KEGG pathways related to hypoxia and development of tumors, which strongly suggested the potential roles of the hypoxic TME in the tumorigenesis and progression of GBM, which may provide new evidence for cancer-targeted treatments involving the prognostic HRGs.

Previous studies have investigated the roles of the 5 prognostic HRGs in the development and progression of GBM. H2AFJ, located on chromosome 12, encodes a replication-independent histone that is a variant H2A histone, which is a basic nuclear protein responsible for the nucleosome structure of the chromosomal fiber. Lee et al. [14] reported that H2AFJ could drive mesenchymal transition and TMZ resistance in GBM. RRM2 encodes one of two nonidentical subunits of ribonucleotide reductase. It was reported to promote the tumorigenicity and progression of GBM cells and may also serve as a prognostic biomarker with functional significance in GBM [15, 16]. THBD encodes an endothelial-specific type I membrane receptor that binds thrombin, which thereby results in the activation of protein C and reduces the amount of thrombin generated. Maruno et al. [17] found that the increased expression of THBD was related to the tumor neovascularization and growth of glioma. Moreover, THBD was also reported to be a therapeutic target for GBM in vitro [18]. PDSS1 encodes the enzyme that elongates the prenyl side-chain of coenzyme Q, or ubiquinone, and may be peripherally associated with the inner mitochondrial membrane. Many studies have investigated the roles of coenzyme Q in suppressing the progression and invasion of glioma cells in vitro and sensitizing GBM cells to TMZ and radiation [19, 20]. ID3 encodes a helix-loop-helix (HLH) protein that can form heterodimers with other HLH proteins that are involved in regulating a variety of cellular processes, including cellular growth, differentiation, apoptosis, angiogenesis, and neoplastic transformation [21]. As reported in the literature, ID3 could promote the formation of stem-like cells and tumor angiogenesis in glioma and might serve as a therapeutic target of low grade gliomas [22, 23]. In summary, the five HRGs are not only promising prognostic predictors but also potential molecular therapeutic targets for GBM.

Then, a novel prognostic prediction model based on the expression levels of the abovementioned five HRGs was successfully generated and validated in separate patient populations. The hypoxia signature demonstrated favorable predictive value in stratifying GBM patients into high- and low-risk subgroups that had significantly different OS and PFS outcomes. Hence, more aggressive treatment strategies and closer follow-ups should be applied in GBM patients with high risk scores according to our novel prognostic signature.

Nomograms have been widely used in clinical practice for their intuitive visualization of statistical models and graphical assessment of variables’ importance [24]. To the best of our knowledge, this is the first prognostic nomogram with a hypoxia signature for predicting the survival of GBM patients that was constructed based on large-scale patient populations with long-term follow-up. In terms of the nomogram for OS prediction, we integrated the hypoxia signature and five other independent clinical risk factors to build the prediction model. However, only three independent predictors, including the hypoxia signature, radiotherapy, and IDH mutation status, were finally selected to construct the progression nomogram for PFS prediction. Both calibration plots and ROC curves suggested the robust and reliable predictive performance of the nomograms for OS and PFS prediction in different populations from the TCGA training cohort and CGGA validation cohort. Therefore, our nomograms might be useful tools for assisting physicians in making individualized prognosis predictions, treatment strategies, and follow-up scheduling.

As reported in the literature, GBM is also characterized by an immunosuppressive TME, where GBM cells, especially GBM stem-like cells (GSCs), recruit immunosuppressive cells into the TME by secreting cytokines and chemokines [25]. The tumor-associated immunosuppressive cells were found to promote the malignant phenotype, immune escape and chemoresistance of GBM [26]. Interestingly, the elevated abundances of the tumor-associated immunosuppressive cells were associated with the poor prognosis of GBM patients [27]. By performing the immune infiltration analysis between TILs and the expression, methylation, and CNA of the HRGs, we found that THBD and H2AFJ were significantly correlated with the tumor-associated immunosuppressive cells, thereby promoting the immunosuppressive TME of GBM. Hence, we believe that hypoxia might promote the immunosuppressive microenvironment of GBM mediated by HRGs. In addition, compared with Cluster 1, Cluster 2 patients tend to be more likely to respond to immunotherapy, especially anti-PD1 therapy, in both training and validation cohort. These findings also suggested that expression patterns of hypoxia signature negatively correlated with the likelihood of immunotherapy.

The underlying mechanisms of GBM chemoresistance mediated by the hypoxic TME have not been fully elucidated. Previous studies reported that the multiple drug resistance (MDR) mechanism was activated by hypoxia to protect cancer cells from different drugs [7]. One of the most important MDR mechanisms at the clinical level is the elevated expression and activity of ATP binding cassette (ABC) transporters, which is related to the resistance to multiple chemotherapies [7, 28]. Another candidate mechanism was that hypoxia would enhance the tumourigenic property of GSCs, which is the core subpopulation of GBM cells, and thereby promote the maintenance, aggressiveness and chemoresistance of GSCs [25]. In addition, the inhibition of proapoptotic pathways was also recently investigated to promote GBM chemoresistance [7] In this study, we found that the hypoxic TME might mediate and promote chemoresistance by regulating the expression levels of the corresponding HRGs. Targeted drugs that can regulate the expression levels of these HRGs might be combined with chemotherapy drugs such as TMZ or bevacizumab to possibly improve or even reverse chemoresistance in GBM patients.

In conclusion, by performing a comprehensive multi-omic analysis based on transcriptomic, DNA methylation and CNA patterns, we developed and validated a hypoxic TME gene-based signature that could be applied for subgroup classification, risk stratification, prognosis prediction, and therapeutic targets for GBM patients. Then, prognostic and progression nomograms for OS and PFS prediction were constructed for individualized survival prediction, better treatment decision-making, and follow-up scheduling. Finally, the GSEA, immune infiltration analysis, and chemotherapy resistance analysis of the HRGs were performed to investigate the vital roles of the hypoxic TME in the development, progression, immune responsiveness and chemoresistance of GBM. Our study not only demonstrated HRGs as predictors of prognosis and immunotherapy and chemotherapy effectiveness but also suggested a new treatment strategy to combat chemoresistant GBM by targeting those HRGs on the basis of conventional chemotherapies. Large-scale, multicenter and prospective studies are needed to validate our prediction model in the future.

## MATERIALS AND METHODS

### Data acquisition and processing

The level three RNA sequencing data and corresponding clinical information of a total of 501 GBM patients were downloaded from The Cancer Genome Atlas (TCGA, https://portal.gdc.cancer.gov/) and the Chinese Glioma Genome Atlas (CGGA, http://www.cgga.org.cn) database. Any patients without prognostic information were excluded. The gene expression profiles of 151 TCGA GBM samples were selected as the training cohort and that of 350 CGGA patients as the validation cohort. In addition, the transcriptomic profiles of the GSE45301 and GSE118683 datasets were downloaded from the Gene Expression Omnibus (GEO, http://www.ncbi.nlm.nih.gov/geo/) database, where the gene expression patterns of GBM cell lines were compared between normoxic (20-21% oxygen) and hypoxic (1-1.5% oxygen) culture conditions [10, 29]. We enrolled 3 normoxia (GSM11027 10-GSM1102712) and 3 hypoxia (GSM1102713-GSM1102715) cultured cells from GSE45301; and also enrolled 4 normoxia (GSM3336604, GSM3336606, GSM3336608, and GSM3336610) and 4 hypoxia (GSM3336605, GSM3336607, GSM3336609, and GSM3336611) cultured cells from GSE118683. The 8 samples of GSE118683 were IDH wildtype GBM stem cells. Ethics committee approval for our study was not required because the data were obtained from publicly available databases.

### Identification of GBM-Specific HRGs and enrichment analyses

First, the Molecular Signatures Database (MSigDB), a collection of annotated gene sets, was applied to screen all the known HRGs [30]. A total of 1694 genes in 65 gene sets were selected as HRGs with the following keywords: hypoxia AND Homo sapiens (Supplementary Table 1). Then, the differentially expressed genes (DEGs) between normoxic and hypoxic cultured GBM cells were screened using the ‘edgeR’ package in R 3.5.1 [31]. Adjusted P (adj. P) values were applied to correct the false positive results by using the default Benjamini-Hochberg false discovery rate (FDR) method. Adj. P value < 0.01 and |fold change (FC)| > 2 were considered as the cutoff criteria for determining DEGs [32]. The dysregulated genes of GSE45301 and GSE118683 in hypoxia were visualized by volcano plots. Finally, the genes in the intersection of the HRGs and the DEGs of GSE45301 and GSE118683 were considered the GBM-specific HRGs for further analysis and were displayed by a Venn diagram.

Then, functional and pathway enrichment analyses for the GBM-specific HRGs were performed by using the Database for Annotation, Visualization and Integrated Discovery (DAVID, http://david.ncifcrf.gov/) database [33]. Gene Ontology (GO) analyses, including the biological process (BP), cellular component (CC) and molecular function (MF) categories, were used for functional annotation, and Kyoto Encyclopedia of Genes and Genomes (KEGG) was used for pathway enrichment analysis. P < 0.05 was considered statistically significant.

### Unsupervised consensus clustering of GBM patients based on the HRGs

Unsupervised consensus clustering, a k-means machine learning algorithm, was applied to explore a novel molecular classification of GBM patients based on the expression patterns of the HRGs using the ‘ConsensusClusterPlus’ package [34]. The clustering procedure with 1000 iterations was performed by sampling 80% of the data in each iteration. The optimal number of clusters was comprehensively determined by the relative change in the area under the cumulative distribution function (CDF) curves, the proportion of ambiguous clustering (PAC) algorithm, and also the consensus heatmap. Then, the cluster quality measures called the “in-group proportion” (IGP) was applied to verify the similarities between different clusters in other independent datasets by using the ‘clusterRepro’ package [35]. Next, Kaplan-Meier (K-M) survival analysis was performed to evaluate the prognosis of different clusters. The distributions of the clinicopathological factors between different clusters were also analyzed to further explore the associations between the HRG-based molecular classification and clinical features of GBM.

### Generation and validation of the prognostic risk score model (hypoxia signature) based on the HRGs

The associations between the expression levels of the HRGs and patients’ OS were first assessed by the univariate Cox regression analysis in the TCGA training cohort. The prognosis-related genes with a P value < 0.05 were further screened by the least absolute shrinkage and selection operator (LASSO) method and multivariate Cox regression analysis. Then, the risk score model based on the HRGs was constructed for predicting the prognosis of GBM patients [36]. Risk score= Exp (Gene_1_) × β_1_ + Exp (Gene_2_) × β_2_ +…+ Exp (Gene_n_) × β_n_; where “Exp” represents the expression level of the gene, and “β” represents the regression coefficient of each gene calculated by the multivariate Cox regression analysis [37]. Then, the prognostic risk score of each patient was calculated according to the formula. All TCGA GBM patients were stratified into a low-risk (low risk score) group and a high-risk (high risk score) group according to the median value of the risk score. K-M survival curve analysis was performed to estimate the OS and PFS of the high-risk and low-risk patients, and the survival differences were evaluated by the two-sided log-rank test. The prognostic performance was evaluated by Harrell's concordance index (C-index) and time-dependent receiver operating characteristic (ROC) curve analysis within 0.5, 1 and 3 years to evaluate the predictive accuracy of the HRG-based prognostic model using the ‘survcomp’ and ‘survivalROC’ packages in R [24, 38]. Both the C-index and area under the curve (AUC) range from 0.5 to 1, with 1 indicating perfect discrimination and 0.5 indicating no discrimination. Finally, the prognostic models constructed by the TCGA training cohort were further validated by the CGGA GBM cohort in a similar way.

In addition, univariate and multivariate Cox regression analyses were performed in the TCGA training cohort and CGGA validation cohort in order to determine whether the predictive power of the hypoxia signature could be independent of other clinicopathological parameters, including age, sex, Karnofsky Performance Status (KPS) score, pharmacotherapy, radiotherapy, surgery, isocitrate dehydrogenase (IDH) mutation status, O6-methylguanine-DNA-methyltransferase (MGMT) promoter methylation status, telomerase reverse transcriptase (TERT) promoter mutation status, B-Raf proto-oncogene (BRAF) mutation status, X-linked alpha thalassemia mental retardation syndrome gene (ATRX) mutation status, epidermal growth factor receptor (EGFR) mutation status, and 1p/19q status.

### Construction and validation of nomograms with the hypoxia signature for predicting OS and PFS

All the independent prognostic parameters that were identified by the univariate analysis and the following multivariate Cox regression analysis were used to construct nomograms to evaluate the 0.5-, 1-, and 3-year OS and PFS probabilities for TCGA GBM patients using the ‘rms’ package. The discrimination performance of the nomograms for prognosis was quantitatively assessed by the C-index and ROC curve analysis [24]. The calibration plots at 0.5, 1, and 3 years were also constructed to graphically evaluate the discriminative ability of the nomograms [38]. Finally, the nomograms for OS and PFS prediction were externally validated by the CGGA GBM cohort.

### GSEA

Setting the expression level of a gene as the population phenotype, GSEA (http://software.broadinstitute.org/gsea/index.jsp) was performed to identify the related KEGG pathways and molecular mechanisms of the HRGs in the hypoxia signature [30]. Enriched gene sets with a nominal P value < 0.05 and an FDR q value < 0.25 were considered statistically significant.

### Immune infiltration analysis of the HRGs

An integrated repository portal for tumor-immune system interactions (TISIDB, http://cis.hku.hk/TISIDB/) is a web portal for tumor and immune system interactions across human cancers that integrates multiple heterogeneous data types [39]. The immune-related signatures of 28 types of tumor-infiltrating lymphocytes (TILs), originating from Charoentong's study, were composed of 3 CD8^+^ T-cell subsets [e.g., activated (Act) CD8, central memory T (Tcm CD8), and effector memory T (Tem CD8) cells], 9 CD4^+^ T-cell subsets [e.g., Act CD4, Tcm CD4, Tem CD4, follicular T-helper (Tfh), gamma delta T (Tgd), T-helper 1, 2, 17 (Th1, Th2, Th17), and regulatory T (Treg) cells], 3 B-cell subsets [e.g., Act B, immature (Imm) B, and memory (Mem) B cells], and 13 innate immune cells, including natural killer (NK) cell subsets [e.g., NK, CD56bright and CD56dim cells], myeloid-derived suppressor cells (MDSCs), NKT, Act dendritic cells (DCs), plasma DCs (pDCs), immature DCs (IDCs), macrophages, eosinophils, mast cells, monocytes, and neutrophils [40]. The relative abundances of TILs were inferred by using gene set variation analysis (GSVA) based on gene expression profiles of GBM. The correlations between TILs and the expression, methylation, and CNA of the HRGs were determined by Pearson’s correlation test. A P value < 0.05 and correlation coefficient > 0.3 or < -0.3 was considered significantly correlative. Then, the regulatory networks between TILs and HRGs were visualized by Cytoscape.

### Predictions of immunotherapy response of the GBM patients

Tumor Immune Dysfunction and Exclusion (TIDE; http://tide.dfci.harvard.edu/) model was a computational method, which integrated the expression signatures of T cell dysfunction and T cell exclusion to model tumor immune evasion [41]. The clinical response of immune checkpoint blockade (ICB) could be predicted by TIDE algorithm based on pre-treatment tumor profiles. Then, an unsupervised subclass mapping method (SubMap; https://cloud.genepattern.org/gp/) was further applied to predict the ICB response of the GBM patients in different clusters based on the novel HRG-based classification [42].

### Chemotherapy resistance analysis of the HRGs

Chemotherapy responsiveness can be influenced by the expression patterns of some key genes, which may serve as potential biomarkers for drug screening. The Cancer Therapeutics Response Portal (CTRP) database, which provides information on 481 small-molecule anticancer drugs across different human cancer cell lines, was used to analyze the correlations of gene expression and drug sensitivity [43]. The correlation analyses between gene expression levels and the area under the dose-response curve values for drugs were performed by the Spearman correlation test. A P value < 0.05 and correlation coefficient > 0.2 or < -0.2 was considered significantly correlative. Positive correlation coefficients indicate that high gene expression is related to drug resistance, while negative correlation coefficients indicate that high gene expression is related to drug sensitivity. All statistical analyses in this study were conducted using R version 3.5.1, and a two-tailed P value < 0.05 was considered statistically significant. Hazard ratios (HRs) and 95% confidence intervals (CIs) were reported if necessary.

## Supplementary Material

Supplementary Figures

Supplementary Table 1

Supplementary Table 2
