# Supplementary material for: A novel hypoxic tumor microenvironment signature for predicting the survival, progression, immune responsiveness and chemoresistance of glioblastoma: a multi-omic study
Source: Aging (Albany NY). 2020 Aug 28;12(17):17038–61. doi: 10.18632/aging.103626 (PMC7521504; doi:10.18632/aging.103626)
Supplement: Supplementary Figures [file aging-12-103626-s003..pdf]

SUPPLEMENTARY FIGURES

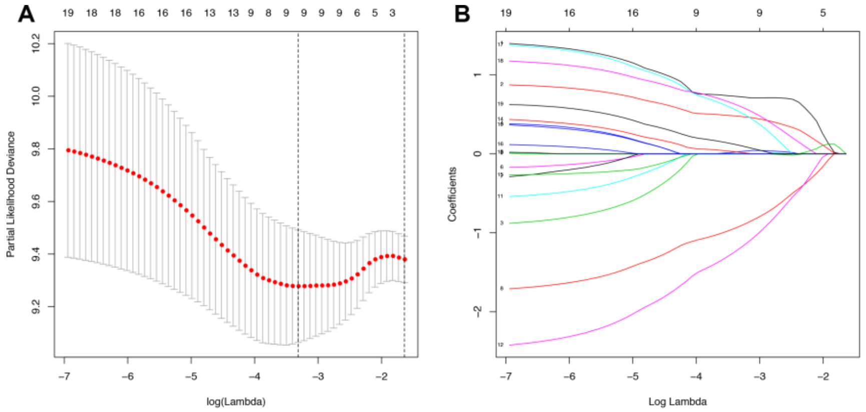

C

id	coef	HR	HR.95L	HR.95H	pvalue
**THBD**	0.894339401	2.445719614	1.294713583	4.619975033	5.85E-03
**ID3**	-1.298174828	0.273029665	0.126267696	0.590374263	9.69E-04
**PDSS1**	-1.781909375	0.168316461	0.049157698	0.576317281	4.55E-03
**H2AFJ**	1.207776337	3.346035918	1.140211713	9.819190799	2.79E-02
**RRM2**	1.132934412	3.104753773	1.449709028	6.649262578	3.55E-03

Supplementary Figure 1. The prognosis-related HRGs were screened by the LASSO and multivariate Cox regression analysis. (**A**) Optimal parameter (lambda) selection in the LASSO model used ten-fold cross-validation via minimum criteria. The partial likelihood deviance (binomial deviance) curve was plotted versus log(lambda). Dotted vertical lines were drawn at the optimal values by using the minimum criteria and the I standard error of the minimum criteria. (**B**) The coefficient profile plot was produced against the log(lambda) sequence. Vertical line was drawn at the value selected using ten-fold cross-validation, where optimal lambda resulted in ten features with nonzero coefficients. (**C**) Following LASSO regression analysis, the five HRGs identified by multivariate Cox regression analysis.

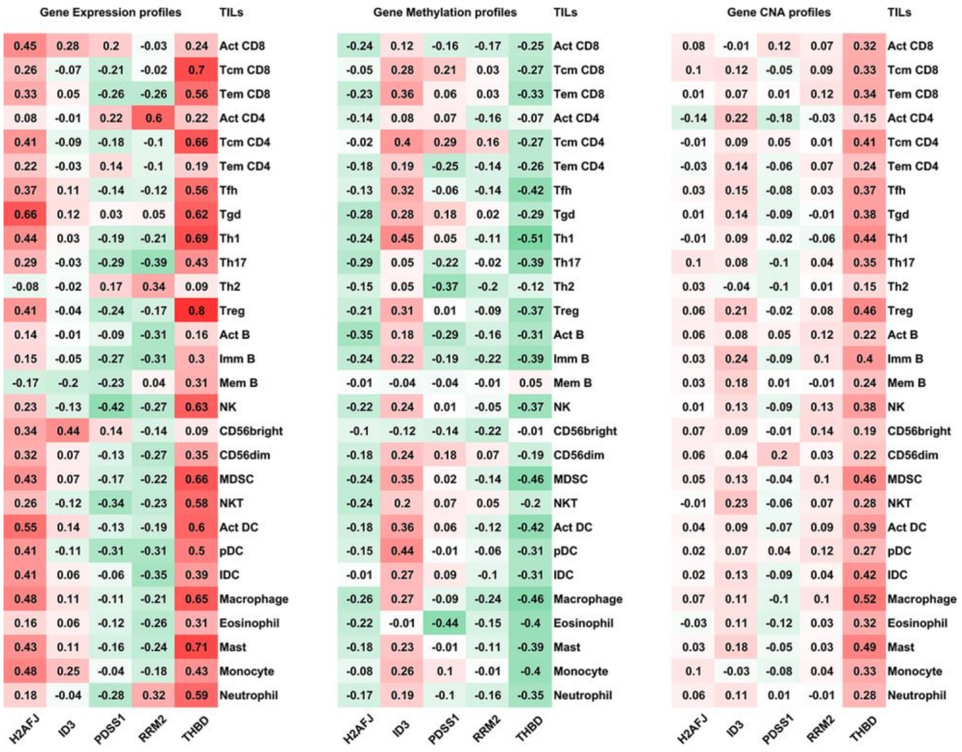

Supplementary Figure 2. Immune infiltration analysis of the five HRGs. The correlations between the abundance of tumor-infiltrating lymphocytes (TILs) and the expression (left panel), methylation (middle panel), and copy number alteration (CNA, right panel) levels of HRGs.

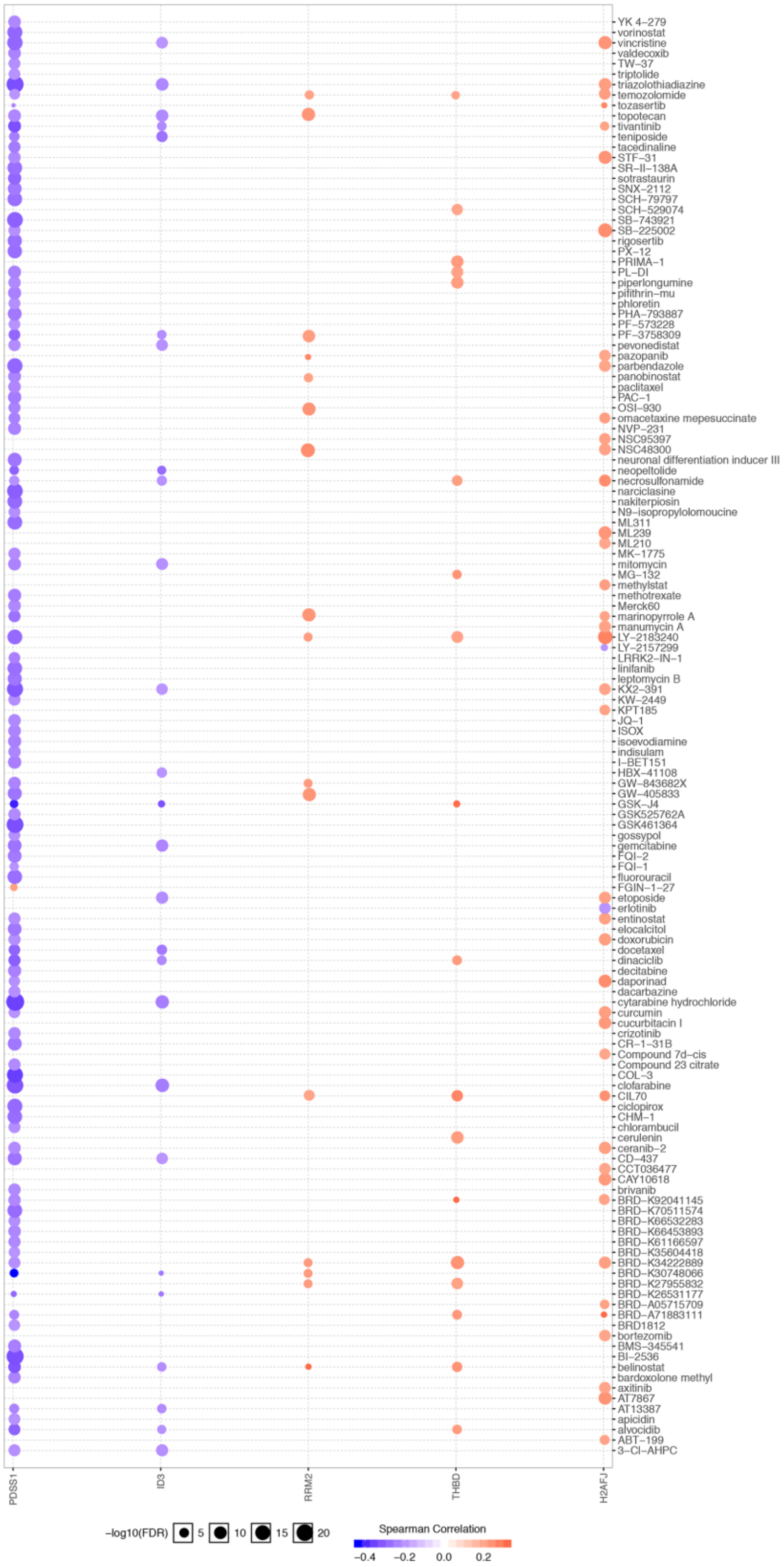

Supplementary Figure 3. The correlation analyses between expressions of the 5 HRGs and the area under the dose-response curve values for drugs. A P value < 0.05 and |correlation coefficient| > 0.2 was considered significantly correlative. The red dots represent positive correlation, which indicates that high expression of the gene is resistant to the drugs. The blue dots represent negative correlation, which indicates that high expression of the gene is sensitive to the drugs.
